# Supplementary material for: Nextstrain automates real-time phylogenetic analysis of open data for endemic and emerging pathogens
Source: Bioinformatics. 2026 Sep 25;42(10):btag705. doi: 10.1093/bioinformatics/btag705 (PMC13661271; doi:10.1093/bioinformatics/btag705)
Supplement: Supplementary file 1 [file btag705_supplementary_data.pdf]

Supplementary Information

Nextstrain automates real-time phylogenetic analysis of open data for endemic and emerging pathogens

Kimberly R. Andrews, Jennifer Chang, Cornelius Roemer, James Hadfield, Victor Lin, Anderson F. Brito, Richard Olumide Daodu, Isabel A. Joia, Kathryn Kistler, Allison Li, Louise H. Moncla, Miguel I. Paredes, Denise Kühnert, Laura Marcela Torres, Laura Voitl, Ivan Aksamentov, Emma B. Hodcroft, John Huddleston, John T. McCrone, John S. J. Anderson, Thomas R. Sibley, Jover Lee, Richard A. Neher, Trevor Bedford

Supplementary Table S1

Table S1. Scaling of workflow run time and memory, and visualization performance across dataset sizes of SARS-CoV-2. This is running the standard "ncov" workflow at `https://github.com/nextstrain/ncov` to produce a single build target, namely `ncov/open/global/all-time/`. This uses a 100k pre-filtered dataset to avoid the overhead of subsampling from the ∼9M publicly available genomes. All runs were on a single AWS c7a instance with 2 cores allocated to the workflow to equilibrate performance while providing enough memory for the large datasets to complete.

Tips	Total wall	Tree	Refine	Peak RAM	JSON	Page load	Recolor
500	4.4 min	32 sec	2.5 min	0.6 GB	1.0 MB	144 ms	63 ms
1000	8.6 min	1.6 min	5.0 min	0.9 GB	1.9 MB	190 ms	108 ms
2000	20 min	6.9 min	10 min	2.1 GB	3.5 MB	267 ms	216 ms
5000	74 min	39 min	28 min	7.3 GB	7.6 MB	487 ms	420 ms
10,000	3.6 hr	2.3 hr	1.0 hr	17 GB	15 MB	842 ms	603 ms
20,000	11.2 hr	8.6 hr	2.2 hr	45 GB	28 MB	1461 ms	885 ms

Supplementary Text

Development of the Nextstrain ecosystem since 2018

The initial Nextstrain publication (Hadfield et al., 2018) described the platform shortly after its inception when it comprised the Augur analysis pipeline and the Auspice visualization application applied to a small number of centrally maintained pathogen builds. The modular Augur architecture that underlies the workflows described here was itself introduced at Augur version 3.0.0 in September 2018, and the ecosystem has since expanded substantially in both capability and scope. We provide a non-exhaustive summary of major developments below, organized by component.

Augur

Augur was refactored from a largely monolithic prepare-and-process pipeline into a modular toolkit (Huddleston et al., 2021) of composable subcommands (e.g., filter, align, tree, ancestral) orchestrated by Snakemake, beginning at version 3.0.0 in September 2018. This composable design is the basis of the workflows described here. This toolkit saw continual feature additions with improvements to subsampling, merging data sources, VCF inputs, etc. A dedicated `augur curate` suite for standardizing and harmonizing metadata was introduced at version 18.2.0 in November 2022 and expanded substantially thereafter; this suite now underpins the metadata curation performed in the ingest workflow.

Nextclade

Nextclade (Aksamentov et al., 2021) did not exist at the time of the 2018 publication (version 1.0.0 was released in June 2021). It performs reference-based alignment, sequence quality control, clade and lineage assignment, mutation calling relative to a reference sequence and clade founders, and is available as both a web application and a command-line tool. Its development enabled the standardized quality-control and clade-calling step now incorporated into our ingest workflows.

Auspice

Auspice has grown from simply a geographic map and phylogenetic tree viewer into a versatile analysis tool for phylodynamic data. New visualisations include a genomic diversity/entropy panel, clade frequency plots, second-tree and tanglegram views, and a measurements panel for displaying titer or deep mutational scanning data (Lee et al., 2023). Auspice also gained Narratives, which pair explanatory prose with linked views into the underlying data that update automatically as the reader moves through the narrative (Hadfield et al., 2019). These changes have necessitated a new v2 dataset schema shared with `augur export v2`.

Nextstrain CLI and runtimes

The `nextstrain` command-line tool was developed to make installation and execution portable and reproducible across computing environments. It supports multiple runtimes, including Docker, Conda, ambient, and Singularity/Apptainer, as well as job submission to AWS Batch. This infrastructure supports the more computationally demanding workflows described here.

nextstrain.org and data sharing

Capabilities for sharing data and results have expanded well beyond centrally hosted builds. Nextstrain Groups allow institutions and public health agencies to share datasets publicly or privately; Community sharing renders datasets directly from any GitHub repository; and a RESTful API at `https://data.nextstrain.org` provides programmatic access to curated sequences, metadata, and analysis outputs, including the per-pathogen data files now surfaced at `https://nextstrain.org/pathogens/files`. Finally, versioned dataset

access is available for our core builds to enable retrospective viewing of analyses from any point in time.

Standardized, automated pathogen workflows

The standardized per-pathogen repository structure that is the primary subject of this paper was developed since our prior publication. Key features of this structure include separate ingest, Nextclade, and phylogenetic Snakemake workflows, shared curation tooling, and GitHub Actions automation producing continually updated results. The separation of individual pathogen builds into dedicated repositories began in September 2018, and the automated, continually updated workflow architecture described here has been built out in the years since.

Supplementary References

I. Aksamentov et al. Nextclade: clade assignment, mutation calling and quality control for viral genomes. *J. Open Source Softw.*, 6(67):3773, Nov. 2021.

J. Hadfield et al. Nextstrain: real-time tracking of pathogen evolution. *Bioinformatics*, 34 (23):4121–4123, Dec. 2018.

J. Hadfield et al. Twenty years of west nile virus spread and evolution in the americas visualized by nextstrain. *PLoS Pathog.*, 15(10):e1008042, Oct. 2019.

J. Huddleston et al. Augur: a bioinformatics toolkit for phylogenetic analyses of human pathogens. *J. Open Source Softw.*, 6(57):2906, Jan. 2021.

J. Lee et al. Joint visualization of seasonal influenza serology and phylogeny to inform vaccine composition. *Front. Bioinform.*, 3:1069487, Mar. 2023.
